# Saliva Gene Promoter Hypermethylation as a Biomarker in Oral Cancer

**DOI:** 10.3390/jcm10091931

**Published:** 2021-04-29

**Authors:** Óscar Rapado-González, José Luis López-Cedrún, Rafael López-López, Ana María Rodríguez-Ces, María Mercedes Suárez-Cunqueiro

**Affiliations:** 1 Department of Surgery and Medical-Surgical Specialties, Medicine and Dentistry School, Universidade de Santiago de Compostela, 15782 Santiago de Compostela, Spain; oscar.rapado@rai.usc.es (Ó.R.-G.); anarces96@gmail.com (A.M.R.-C.); 2 Translational Medical Oncology Group (Oncomet), Health Research Institute of Santiago (IDIS), Complexo Hospitalario Universitario de Santiago de Compostela (SERGAS), 15706 Santiago de Compostela, Spain; rafael.lopez.lopez@sergas.es; 3 Centro de Investigación Biomédica en Red en Cáncer (CIBERONC), Instituto de Salud Carlos III, 28029 Madrid, Spain; 4 Department of Oral and Maxillofacial Surgery, Complexo Hospitalario Universitario de A Coruña (SERGAS), 15006 A Coruña, Spain; lopezcedrun@centromaxilofacial.com

**Keywords:** DNA methylation, epigenetics, saliva, oral cancer, biomarker, tumor-suppressor genes

## Abstract

Oral carcinogenesis is a multistep process characterized by a summation of multiple genetic and epigenetic alterations in key regulatory genes. The silencing of genes by aberrant promoter hypermethylation is thought to be an important epigenetic event in cancer development and progression which has great potential as a biomarker for early diagnosis, tumor molecular subtyping, prognosis, monitoring, and therapy. Aberrant DNA methylation has been detected in different liquid biopsies, which may represent a potential alternative to solid biopsies. The detection of methylated genes in saliva may have clinical application for noninvasive oral cancer screening and early diagnosis. Here, we review the current evidence on gene promoter hypermethylation in saliva.

## 1. Introduction

Oral cancer is a multifactorial disease that arises as a result of the interaction of lifestyle, environmental, genetic and epigenetic factors. The most important risk factors include tobacco and alcohol, whose synergistic consumption has been associated with an increased risk [1]. Human papillomavirus infection has also been identified as a risk factor [2]. According to GLOBOCAN 2018 estimates, oral cancer represents one of the most common types of cancer worldwide with approximately 355,000 new cases and 178,000 deaths annually [3]. Despite advances in clinical research, most patients are still diagnosed with advanced-stage disease, resulting in a poor prognosis [4].

From a molecular standpoint, oral carcinogenesis is a multistep process characterized by a summation of multiple genetic and epigenetic alterations in key regulatory genes leading to the transformation of normal oral epithelial cells into oral squamous cell carcinoma. Epigenetic alterations include the key process of DNA methylation, histone covalent modifications, chromatin remodeling, and the effect of noncoding RNAs in gene expression [5]. DNA methylation is one of the most intensively studied epigenetic modifications of the human genome that is involved in the regulation of many cellular processes including embryonic development, transcription, chromatin structure, X chromosome inactivation, genomic imprinting and chromosome stability [6]. DNA methylation consists of the reversible addition of a methyl group to the carbon-5 position of the cytosine ring within CpG dinucleotides to form 5-methylcytosine (5-mC). The transferring of the methyl group from S-adenosylmethionine as the donor molecule to the cytosine base is catalyzed by a family of enzymes called DNA methyltransferases. This process usually occurs in CpG islands, rich in CpG dinucleotides and often located in gene promoter regions that are normally unmethylated [7]. Widespread changes in DNA methylation have been identified in all types of tumors which are characterized by exhibiting a global hypomethylated genome that is often accompanied by focal hypermethylation of the CpG islands in the promoter regions of the genes and first exon [8]. Hypermethylation within the promoter region has been associated with the transcriptional silencing of certain tumor-suppressor genes, whereas global hypomethylation of repetitive sequences and transposable elements within the genome induces genomic instability and activates oncogene transcription [9].

In recent years, numerous studies have focused on the detection of aberrant promoter methylation in a wide variety of tumors, such as lung cancer [10], colorectal cancer [11] or head and neck cancer [12]. Scientific evidence has shown that the methylation profile of gene promoters is different for each human cancer, allowing the identification of cancer-specific hypermethylation patterns [13]. Currently, pan-cancer methylation studies are being undertaken to identify common and tissue-specific DNA methylation patterns across multiple cancer types to better understand the mechanisms of tumorigenesis [14]. Furthermore, gene promoter hypermethylation is thought to be an important early event in cancer development and progression which has great potential as a biomarker for early diagnosis, tumor molecular subtyping, prognosis, monitoring and therapy. Aberrant DNA methylation can be detected in different types of body fluids, such as blood [15], sputum [16], bronchial lavage fluid [17], urine [18] or saliva [19]. Nowadays, saliva represents a potential alternative to solid biopsy both in oral tumors and tumors distant to the oral cavity due to its noninvasive collection and its composition enriched with tumor biomarkers such as noncoding RNAs, proteins, mRNA, and genomic DNA [20,21,22]. Although the oral cavity is easily accessible, the tissue biopsy may not adequately represent the tumor heterogeneity. Furthermore, molecular alterations may be identified in saliva before the tumor is clinically detectable, allowing for early disease detection. New clinical tools are necessary to allow the screening in high-risk populations and oral cancer diagnosis in a cost-effective manner. In this sense, the detection of DNA methylation in saliva has emerged as a potential method for the early diagnosis of head and neck tumors. Numerous oral cancer studies have evaluated the methylation profile of oral exfoliated cells collected from saliva (i.e., salivary oral rinses or whole saliva) [19,23,24,25]. The presence of salivary DNA promoter hypermethylation could be a potential clinical biomarker for the development of oral cancer.

Here, we provide an overview of the current evidence regarding gene promoter hypermethylation in saliva for oral cancer screening, diagnosis and prognosis.

## 2. Saliva Hypermethylation: An Early Event in Oral Cancer

Promoter hypermethylation can be found early in tumorigenesis involving loss of cell cycle control, altered function of transcription factors, altered receptor function, disruption of normal cell–cell and cell–substratum interaction, inactivation of signal transduction pathways, loss of apoptotic signals and genetic instability [26]. The characterization of the epigenetic alterations underlying oral carcinoma progression (normal mucosa-basal cell hyperplasia-dysplasia-carcinoma in situ- invasive carcinoma) is key for understanding the dynamic of the epigenetic landscape during the progression from oral precancer to cancer. Changes in DNA methylation patterns could be useful for predicting the rate and likelihood of malignant transformation, thus representing a potential biomarker for high-risk lesions [27]. Several studies have investigated the methylation profile of various tumor suppressor and DNA repair genes in oral-precancer. *DAPK*, *MGMT*, *p16* or *ECAD* genes have been identified as hypermethylated markers in oral leukoplakia [28,29]. Interestingly, Bhatia et al. compared the methylation status of *p16* and *MGMT* in tissue and blood from oral premalignant lesions, oral cancer, and healthy controls. Significant promoter methylation of *MGMT* and *p16* genes was detected both in tissue and blood from oral premalignant patients as well as in oral cancer patients. Furthermore, *MGMT* and *p16* genes showed a downregulated expression both in premalignant oral lesions and oral cancer, suggesting their potential involvement in the progression to malignancy [30]. Two longitudinal studies reported that *p16* hypermethylation in precancerous lesions could be a predictor of malignant transformation [31,32]. However, promoter methylation of *MGMT*, *CYGB*, and *CCNA1* was not correlated with malignant progression [31]. In another study, *DAPK* promoter hypermethylation showed similar frequency in tissue and blood from oral precancer (19.5% vs. 20.9%, respectively) and oral cancer (46.9% vs. 52.2%, respectively). Importantly, a significant correlation was found between tissue and blood *DAPK* hypermethylation, demonstrating the potential of methylated genes as early biomarkers in liquid biopsies [33]. Moreover, this study also detected *DAPK* hypermethylation at a very low level in salivary oral rinse samples. Previously, López et al. reported high methylation of *p16* and *MGMT* in salivary oral rinses of patients with homogeneous oral leukoplakia. Interestingly, oral leukoplakia patients with a previous history of one or more oral squamous cell carcinomas showed an increased *p14* promoter hypermethylation, suggesting that inactivation *p14* increases the risk of oral cancer transformation [34]. In addition, *p16* promoter methylation was observed in 50% of saliva samples from patients with oral submucosal fibrosis. This percentage increased to 93.3% when *p16* methylation was analyzed in buccal cells obtained by cytobrush. The fact that no hypermethylation was found in healthy controls suggests *p16* as an early indicator of carcinogenic activity [35]. Recently, Cheng et al. evaluated the methylation levels of *ZNF582* and *PAX1* genes in salivary oral rinses from normal controls, oral potentially malignant disorders (hyperplasia/hyperkeratosis, mild dysplasia, moderate dysplasia, and severe dysplasia), and oral cancer. The positive rates of both genes (*ZNF582* and *PAX1*) gradually increased in line with oral lesion severity, showing a marked increase from mild dysplasia to moderate dysplasia. Moreover, salivary methylated *ZNF582* and *PAX1* could discriminate moderate dysplasia and other several oral lesions from normal controls as well as hyperplasia/hyperkeratosis and mild dysplasia lesions [36].

Overall, these data support the very early occurrence of promoter hypermethylation in the process of oral carcinogenesis and, therefore, a progression model based on salivary methylated genes could have great clinical value for predicting the risk of oral malignant transformation (Table 1).

## 3. Saliva Hypermethylation as Diagnostic Biomarker in Oral Cancer

The first study to evaluate promoter hypermethylation in salivary DNA from oral cancer patients was carried out in 2007. Viet et al. analyzed the promoter hypermethylation of five genes (*APC*, *ECAD*, *MGMT*, *p15*, and *p16*) in oral cancer, oral dysplasia, and normal controls. Methylation of *p16* was detected in 35% of oral cancer/dysplasia patients whereas *MGMT* and *p15* methylation was detected in 29%, *APC* in 14%, and *ECAD* in 7%. A high level of agreement was found between matched tissue and salivary DNA samples, presenting a correlation of 87.5% for *p16* and *ECAD*, and 62.5% for *MGMT* and *p15* [37]. In another study, this research group identified 41 gene loci of 34 methylated genes (*ADCYAP1*, *AGTR1*, *BMP3*, *CEBPA*, *EPHA5*, *ERBB4*, *ESR1*, *ETV1*, *EYA4*, *FGF3*, *FGF8*, *FLT1*, *GARB3*, *GALR1*, *GAS7*, *HLF*, *IHH*, *IL11*, *INSR*, *IRAK3*, *KDR*, *NOTCH3*, *NTRK3*, *p16*, *PKD2*, *PTCH2*, *PXN*, *RASGRF1*, *TFPI2*, *TMEFF1*, *TNFSF10*, *TWIST1*, *WNT2*, and *WT1*) in preoperative saliva and tissue from oral squamous cell carcinoma patients using a methylation array which included 1505 CpG loci covering 807 genes. Interestingly, various diagnostic panels were performed using combinations of 4 to 10 genes, showing sensitivity values ranging from 62% to 77% and specificity values ranging from 88% to 100% [24]. A larger genomewide DNA methylation study comprising 27,578 CpG sites performed by Guerrero-Preston et al. revealed 301 potential tumor suppressor genes significantly hypermethylated in oral squamous cell carcinoma vs. normal tissues, 92 genes hypermethylated in leukoplakia vs. normal mucosa, and 143 hypermethylated genes in tumor vs. leukoplakia tissue. Based on multiple selection criteria, a total of 8 genes (*EDNRB*, *HOXA9*, *GATA4*, *NID2*, *MCAM*, *KIF1A*, *DCC*, and *CALCA*) were selected for validation by quantitative methylation-specific PCR (qMSP) in 24 oral cancer and 12 normal oral mucosal tissue samples from discovery cohort. Differential methylation between cases and controls was observed for *EDNRB, HOXA9*, *GATA4*, *NID2*, *KIF1A*, and *DCC* genes. The validation of HOXA9 and NID2 in an independent cohort including 55 tumors and 37 normal tissues showed the high diagnostic accuracy of *HOXA9* (85% sensitivity and 97% specificity) and *NID2* (87% sensitivity and 95% specificity) for discriminating head and neck cancer patients, attaining an AUC value of 0.97 when both genes were combined. Furthermore, the promoter methylation status of *HOXA9* and *NID2* was evaluated in salivary oral rinses from 16 oral cancer, 16 oropharyngeal cancer, and 19 healthy controls. Receiver operating characteristic (ROC) curve analysis yielded an AUC of 0.75 for *HOXA9* and an AUC of 0.73 for *NID2* for discriminating oral cancer patients from normal controls. Moreover, the combination of two saliva genes (*HOXA9* + *NID2*) improved this discriminatory power (AUC of 0.77). However, both genes showed a decrease in sensitivity for detecting oropharyngeal cancer patients by saliva, which could be due to the different etiology of these tumors where the human papillomavirus infection is the main risk factor. The authors also suggested that saliva could have less contact with tumors located in the oropharynx than those in the oral cavity, thus reducing the number of tumoral cells in saliva collection [23]. Later, Langevin et al. identified and validated a methylation classifier based on 22 CpG islands using oral rinses from 154 oral and pharyngeal cancer patients and 72 healthy controls through the Infinium HumanMethylation450 BeadArray. This saliva-based methylation biomarker panel showed an AUC of 0.92, which indicated its high degree of accuracy for predicting oral and pharyngeal carcinoma [43].

**Figure 1:**
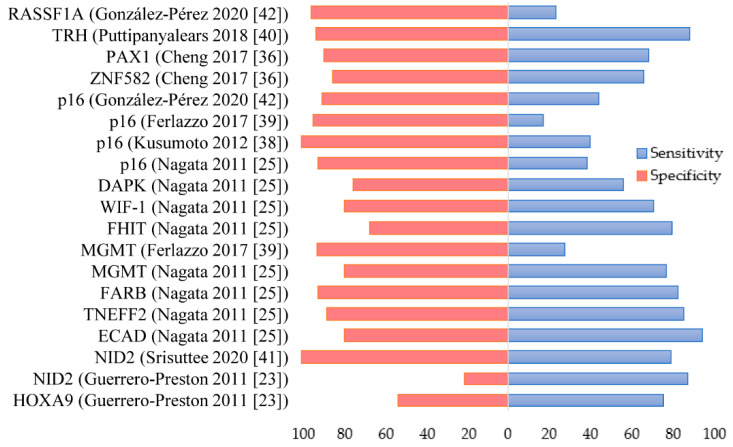
Reported sensitivities and specificities of DNA methylation biomarkers for the detection of oral cancer.

Over the last few years, several DNA-methylation studies in saliva have investigated the promoter hypermethylation status of various genes which had previously been identified as methylated in oral cancer tissue such as *CDKN2A*, *MGMT*, *DAPK1*, *RASSF1A* or *ECAD* (Figure 1). Nagata et al. analyzed the promoter methylation of 13 genes in salivary oral rinses from 34 oral cancer patients and 24 healthy controls, finding significantly higher levels of methylation for 8 genes (*ECAD*, *MGMT*, *DAPK*, *RARβ*, *p16*, *TMEFF2*, *WIF-1*, and *FHIT*) in tumor vs. normal salivary samples. Interestingly, after several saliva gene panels combining 4 genes (*ECAD*, *MGMT*, *RARβ* and *TMEFF2*), it was observed that the salivary 4-gene panel yielded 100% sensitivity and 87.5% specificity whereas 3-gene salivary panels yielded sensitivity and specificity values ranged from 91.2% to 97.1%, and 91.7% to 95.8%, respectively. These results suggest the potential of salivary promoter DNA methylation for detecting oral cancer noninvasively [25]. A frequent event in oral carcinogenesis is the dysregulation of *p16/CDKN2A gene*, a well-recognized tumor suppressor gene in cancer involved in cell-cycle control [44]. Aberrant promoter hypermethylation of *CDKN2A* has been reported in several head and neck cancer studies [45,46,47]. A pilot study performed by Kusumoto et al. analyzed the presence of different epigenetic alterations associated with *CDKN2A* inactivation in salivary oral rinses from 10 oral cancer patients and 3 healthy controls. Both DNA promoter methylation and/or histone modification was detected in all oral cancer patients, while CDKN2A presented negative or low expression in four cases. These findings suggest the involvement of different epigenetic alterations in the regulation of CDK2A expression [38]. In another study, Ferlazzo et al. analyzed *p16* and *MGMT* promoter methylation in saliva from 58 oral cancer patients and 90 controls. The methylation of either the *p16* or *MGMT* promoter regions was significantly higher in oral cancer compared to controls (44.8% vs. 13.4%). In addition, *p16* promoter was significantly methylated more commonly in oral cancer patients with methylenetetrahydrofolate reductase (MTHFR) CT/AC or TT/AA genotype with respect to normal MTHFR genotype. A similar frequency was observed for *MGMT* methylation, although no significant difference was found. These results suggest that MTHFR polymorphisms may have an important role in oral cancer; however, further research is necessary to understand their influence on the gene-specific methylation process [39]. The *MGMT* gene is related to DNA repair and aberrant promoter hypermethylation has been observed frequently in head and neck cancer [47,48]. Recently, Liyanage et al. investigated promoter hypermethylation using a panel of 4 tumor suppressor genes in saliva from 54 oral and 34 oropharynx cancer patients and 60 healthy controls using methylation-specific PCR (MSP) coupled with densitometry analysis. *RASSF1A*, *TIMP3*, and *PCQAP/MED15* showed significant promoter hypermethylation in saliva from oral and oropharynx cancer groups compared to the control group, but no significant methylation was observed for *p16*. Promoter hypermethylation of the *p16* and *RASSF1A* genes was significantly associated with advanced-stage and high-grade oral cancer tumors. In addition, a significant association was observed between *p16*, *RASSF1A*, and *TIMP3* hypermethylation and high-grade oropharynx tumors. Interestingly, *p16* and *RASSF1A* were significantly associated with alcohol and tobacco consumption, whereas the promoter hypermethylation of the four tumor suppressor genes was significantly associated with betel quid chewing. These results suggest that external factors such as tobacco and alcohol may modulate the DNA methylation mechanisms contributing to oral cancer development. Furthermore, the combination of these 4 methylated genes presented a 91.7% sensitivity and 92.3% specificity for oral cancer, and 99.8% sensitivity and 92.1% specificity for oropharynx cancer. These results reflect the high discriminatory power of this salivary promoter hypermethylation gene-panel for both malignancies [19]. The *TIMP3* is an extracellular matrix-bound protein that regulates the activities of matrix metalloproteinases and suppresses cancer cell growth, angiogenesis, migration, and invasion. Promoter methylation of *TIMP3* has been shown to promote oral cancer metastasis [49]. The *PCQAP/MED15* is a transcriptional coactivator mediator essential for the regulated expression of protein-coding genes which have been linked to transforming growth factor-β signaling in head and neck cancer [50]. The *RASSF1A* is one of the most frequently hypermethylated tumor suppressor genes in human cancer that modulates multiple apoptotic and cell cycle checkpoint pathways [51]. In another study investigating two tumor suppressor genes (*p16* and *RASSF1A*), salivary promoter methylation was significantly more frequent in oral cancer patients (44.2% and 23.3%, respectively) compared to healthy controls (10% and 2%, respectively). A high level of specificity was observed when both genes were combined, whereas the sensitivity of methylation detection was 53.5%. *p16* and *RASSF1A* methylation was found to be significantly associated with advanced clinical stages, poorly differentiated tumors, and severe cellular atypia [42].

Recent studies investigating novel methylated tumor-related genes have made use of publicly available methylation microarray data to identify methylation markers for oral cancer diagnosis. Using data from the Gene Expression Omnibus repository, Puttipanyalears et al. identified 27,578 CpG sites and found that site cg01009664 of the *TRH* gene showed the greatest methylation level difference between healthy cells and head and neck cancerous cells. This TRH site-specific methylation was validated in 9 healthy controls and 9 oral cancer patients by pyrosequencing, yielding a methylation percentage of 7% ± 3.43% in healthy cells in contrast to 63% ± 19.81% in cancerous cells. Moreover, high *TRH* methylation was found by quantitative real-time PCR in salivary oral rinses and oral swabs from a discovery cohort comprising 23 oral cancer patients and 33 healthy controls. The validation in oral rinses from 42 oral cancer and 54 healthy controls, demonstrated the high discriminatory power of TRH methylation (AUC = 0.93). Methylation of *TRH* in oral rinses can also discriminate oropharyngeal cancer patients from healthy controls with 82.61% sensitivity and 92.59% specificity (AUC = 0.88). Importantly, this gene presented very similar diagnostic ability when both sample cohorts were considered together (86.15% sensitivity, 89.66% specificity, and AUC = 0.93), indicating the potential of this biomarker for oral cancer detection [40]. A more recent study by the same research group selected another specific CpG site of the *NID2* gene, cg22881914. After initial validation, the methylation of *NID2* was evaluated in salivary oral rinses from 43 oral cancer patients, 40 smokers, and 50 healthy controls by quantitative real-time PCR. High salivary methylation of *NID2* was observed in oral cancer patients whereas no methylation was detected in smokers and healthy controls. In addition, sensitivity increased up to 90.91% when the *NID2* methylation was detected in oral swabs from matched oral cancer patients (n = 22), which could be explained by the larger number of epithelial cells from cancer lesions using oral swabs vs. salivary oral rinses [41].

## 4. Saliva Hypermethylation as Prognostic Biomarker in Oral Cancer

Numerous studies have demonstrated the prognostic value of gene promoter hypermethylation in several types of cancer [52,53,54,55]. However, few studies have evaluated DNA methylation markers in saliva for predicting prognosis in head and neck cancer [56,57]. Promoter hypermethylation of *TIMP3* gene in post-treatment salivary oral rinses has been reported as an independent prognostic factor for local recurrence in head and neck cancer [56]. Focusing on oral cancer, only one study has described the prognostic potential of saliva DNA methylated targets. In this study, a total of 31,038 CpG loci were significantly associated with overall survival by data analysis of 88 oral cancer patients from TCGA cohort. Afterwards, an epigenomewide array using oral rinses from 82 oral cancer patients (for replicating the putative survival-associated GpC loci) revealed 3716 survival-associated CpG loci. Seven CpG loci belonging to *OPCML*, *ADCK4*, *ZFYVE26*, *GABBR1*, *POLR3E*, and *KIF11* genes were selected for validation by pyrosequencing in an independent cohort (*n* = 61). Of these, only the cg21022792 locus located in the body of *GABBR1* was validated as a survival-associated DNA methylation marker, which reflects the possible clinical utility of salivary DNA methylation markers for prognosis in oral cancer [58]. Although further research is required, DNA promoter hypermethylation has the potential to become a saliva-based prognostic marker in head and neck cancer.

## 5. Saliva as a Potential Source to Study DNA Methylation

Saliva has been reported as a source of high-quality DNA for genomic and epigenomic studies [59,60]. Human saliva contains both cellular and extracellular DNA. The latter, coined by our team as salivary cell-free DNA (scfDNA), is a mixture of human and bacterial DNA [61]. Nevertheless, to date only salivary cellular DNA has been used to analyze DNA methylation in oral cancer by various extraction methods (Table 1). Although no oral cancer study has yet analyzed methylation using scfDNA, the epigenetic analysis of scfDNA could provide additional information both in local and distant tumors to the oral cavity.

## 6. Technologies for DNA Methylation Assay

**Figure 2:**
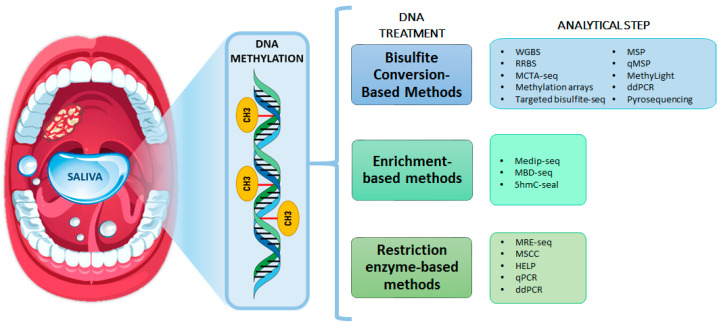
Schematic representation of the technologies for DNA-methylation evaluation.

The methods for the analysis of DNA methylation can be divided into bisulfite conversion-based and nonbisulfite conversion methods (Figure 2). Bisulfite-conversion based methods use a chemical reaction that converts unmethylated cytosine residues to uracil by deamination while methylated cytosines residues remain unchanged. In the present review, bisulfite treatment was the main method used in salivary DNA methylation assays (Table 1). Although bisulfite conversion represents the gold standard for DNA methylation analysis, this technique has several disadvantages such as DNA degradation, the inability to differentiate 5-mC from 5-hydroxymethylcytosine (5-hmC), and limited conversion efficiency that can lead to false-positive and false-negative results [62,63,64]. The analysis of bisulfite-converted DNA can be performed by genomewide and targeted methods. The former includes methods allowing the evaluation of whole methylome such as whole genome bisulfite sequencing (WGBS), reduced-representation bisulfite sequencing (RRBS), methylated CpG tandems amplification and sequencing (MCTA-seq), and methylation array [65]. By contrast, targeted methods focus on the detection of the DNA methylation signal in preselected regions of interest including targeted bisulfite sequencing and PCR-based assays. Targeted bisulfite sequencing techniques represent a cost-efficient method with high depth of sequencing coverage that allow the simultaneous analysis of multiple genomic regions by target PCR amplification or probe hybridization capture. In addition, different PCR-based techniques have been used for evaluating the DNA methylation status at a locus-specific level, such as MSP, qMSP, MethyLight and droplet digital PCR (ddPCR) [66]. Overall, the most frequently used methods for evaluating salivary DNA methylation in oral cancer were MSP and qMSP.

On the other hand, nonbisulfite conversion methods can be divided into antibody enrichment methods and restriction enzyme-based methods. Antibody enrichment methods based on the specificity of antimethylcytosine antibodies or methyl CpG binding proteins to enrich for methylated DNA regions of the genome, such as methyl DNA immunoprecipitation sequencing (MeDIP-seq) and methyl-CpG binding domain protein capture sequencing (MBD-Seq) methods [65,67]. Recently, 5-hmC sequencing methods (5hmC-Seal) have been developed to determine the distribution of hydroxymethylation patterns in the genome [68]. Restriction enzyme-based methods use methylation-sensitive restriction enzymes that can cleave selectively only unmethylated DNA or methylation-insensitive restriction enzymes that can cleave regardless of recognition site methylation status. Using this approach, different techniques can be applied to detect fragmented DNA after digestion such as HpaII-tinny fragment enrichment by ligation-mediated PCR (HELP), methyl-sensitive cut counting (MSCC), methylation restriction enzyme sequencing (MRE-seq), qPCR or ddPCR [69].

## 7. Cell-Type Deconvolution from DNA Methylation

Epigenomewide methylation data obtained from complex biofluids such as saliva or blood comprise signals from the different cell-populations present in the sample. This could result in different epigenetic features and therefore influence genomewide results [70]. To overcome this challenge, various deconvolution approaches have been developed to interrogate cell-type specific methylation, including reference-based and reference-free algorithms. Reference-based deconvolution algorithms depend on reference DNA methylation profiles of cell-types that are present in the sample of interest. However, few studies in saliva have analyzed the methylation profile of salivary cell subtypes [71]. In contrast, reference-free deconvolution algorithms do not require reference data to estimate the putative number and proportion of cell-types. Houseman et al. described a reference-free deconvolution method estimating the proportion of putative cell types defined by their underlying methylomes; the number of these constituent cell types, as well as the extent to which the underlying methylomes reflect specific types of cells [72]. This reference-free method represents a valuable approach in genomewide methylation studies based on samples with cell-heterogeneity and without reference data for cell populations. Future epigenomic profiling of specific salivary cellular populations would make it possible to determine the methylation profile of specific cell types, providing the scientific community with reference data for the correction of genomewide methylation analysis.

## 8. Future Perspectives and Challenges

Currently, the presence of tumor DNA in saliva has emerged as an opportunity for identifying genetic and epigenetic alterations in human cancer. The present review has updated the potential of DNA methylation saliva-based biomarkers for the diagnosis and prognosis of oral cancer. However, the evidence is still limited, and various challenges remain to be addressed before it can be clinically implemented. Firstly, the scientific community should establish standardized protocols for saliva collection and DNA extraction, bisulfite modification, methylation detection methods, and data analysis that allow for reproducibility. Two types of saliva samples were used to detect DNA methylation in the cellular fraction: salivary oral rinses and drool saliva. However, the saliva collection method could affect the concentration of cells and DNA. It is important to note that other factors, such as tumor size and anatomic location, could influence cell shedding into the saliva. Moreover, researchers have used different methods for salivary DNA methylation analysis that could contribute to the variability of results observed in the literature. Secondly, future studies of methylation in saliva should consider the impact of alcohol consumption and smoking as well as other conditions such as diet, sleep deprivation, chronic stress or coexistent diseases. To increase the robustness of the findings, these variables should be included in statistical complex models in combination with phenotype data. Furthermore, studies should be designed with larger sample sizes and likely with a multicenter approach. Thirdly, the majority of the aberrantly methylated genes described in this review had insufficient diagnostic accuracy as single markers. New molecular biology techniques such as next-generation sequencing platforms and digital PCR show improved sensitivity in methylation assays.

Currently, noninvasive early detection using molecular biomarkers in cancer is challenging. DNA methylation is highly stable and robust, making it an attractive biomarker for early detection, assessment of prognosis, prediction of therapy response, and therapy monitoring. DNA methylation-based biomarker tests have demonstrated potential utility in various types of tumors, although only the Epi proColon 2.0 CE test has been approved by the Food and Drug Administration (FDA, USA) for early colorectal cancer detection in liquid biopsy [73]. In this sense, DNA methylation saliva-based tests could represent a potential tool for clinical implementation in oral cancer. Seeing as intra- and intertumoral heterogeneity is recognized as a barrier to the development of tests for clinical implementation, saliva could provide vital additional molecular data to solid biopsy by reflecting landscape heterogeneity. The identification and validation of the methylation profiles based on the promoter CpG island hypermethylation of multiple genes associated with oral cancer or precancer may have potential clinical application for oral cancer detection rather than single-gene alteration. Although some saliva-based methylation biomarker panels have shown high potential for oral cancer diagnosis, further validation is still necessary.

## 9. Conclusions

In summary, salivary DNA methylation holds great promise in oral cancer, but larger studies with a careful methodological design should be carried out. Although the evidence supports the potential clinical utility of salivary DNA methylation in oral cancer, there is still a long way ahead including different research approaches before epigenetic tests can be implemented in clinical oncology.

## Figures and Tables

**Table 1:** Characteristics of the salivary DNA methylation studies on oral cancer and precancer.

Biomarker	Cases (*n*)	Controls (*n*)	Pathology	Type of Controls	Sample Collection	DNA Extraction/DNA Treatment	Method	Se (%)	Spe (%)	Ref.
p16	14	5	OC (SCC) or oral dysplasia	HC	Saliva	Volume: 1 mL Kit: QIAamp Blood; Qiagen/EpiTect Bisulfite; Qiagen	Methylight	35	-	Viet 2007 [37]
MGMT								29	-	
p15								29	-	
APC								14	-	
ECAD								7	-	
AGTR + ESR1 + FLT1 + NOTCH3	13	13/10	OC (SCC)	Postoperatory cancer group/HC	Saliva	Volume: 1 mL Kit: iPrep ChargeSwitch Buccal Cell; Invitrogen/EZ DNA Methylation; Zymo Research	Illumina GoldenGate Methylation Array	63	83	Viet 2008 [24]
GABRB3 + IL11 + INSR + NOTCH3 + NTRK3 + PXN								77	87	
ERBB4+IL11 + PTCH2 + TMEFF1 +TNFSF10 + TWIST1								62	100	
ADCYAP1 + CEBPA + EPHA5 + FGF3 + HLF + IL11 + INSR + NOTCH3								69	96	
AGTR1 + BMP3 + FGF8 + NTRK3								62	87	
ERBB4 + FLT + INSR + IRAK3 + KDR + NTRK + PTCH2 + PXN + RASGRF1 + WT1								69	78	
ESR1 + ETV1 + GAS7 + IL11 + PKD2 + TMEFF1 + WNT2								62	83	
EPHA5 + FGF3 + GALR1 + IL11 + INSR + KDR + p16								62	83	
AGTR1 + ERBB4 + EYA4 + FLT1 + IHH + NTRK3 + MNTRK3 + TFP12								62	78	
HOXA9	16	19	OC (SCC)	HC	Oral rinse (20 mL NaCl)	Volume: Kit: Phenol-chloroform extraction/EpiTect Bisulfite; Qiagen	qMSP	75	53	Guerrero-Preston 2011 [23]
NID2								87	21	
HOXA9 + NID2								50	90	
ECAD	34	24	OC (SCC)	HC	Oral rinse (20 mL NaCl, 30–60 s)	Volume: 5 mL Kit: DNeasy Blood and Tissue; Qiagen/EpiTect Bisulfite; Qiagen	MSP	94.1	79.2	Nagata 2012 [25]
TNEFF2								85.3	87.1	
RARB								82.4	91.7	
MGMT								76.5	79.2	
FHIT								79.4	66.7	
WIF-1								70.6	79.2	
DAPK								55.9	75.0	
p16								38.2	91.7	
HIN-1								29.4	91.7	
TIMP3								23.5	95.8	
p15								64.7	62.5	
APC								52.9	62.5	
SPARC								41.2	66.7	
ECAD + TMEFF2 + RARB + MGMT								100	87.5	
ECAD + TMEFF2 + MGMT								97.1	91.7	
ECAD + TMEFF2 + RARB								94.1	95.8	
ECAD + RARB + MGMT								91.2	91.7	
DAPK	77	32	OC (SCC)	Oral precancer	Oral rinse (NaCl)	Volume: Kit: Phenol-chloroform extraction/Bisulfite solution	qMSP	3.4	97.2	Liu 2012 [33]
p16	10	3	OC (SCC)	HC	Oral rinse (16 mL NaCl, 30 s)	Volume: 3 mL Kit: Methylamp Whole Cell Bisulfite Modification; Epigentek	MSP	40	100	Kusumoto 2012 [38]
p16	30	30	OSMF	HC	Oral rinse (20 mL 0.9% NaCl, 60 s)	Volume: Kit: QIAamp DNA Mini/EpiTect Bisulfite; Qiagen	qMSP	50	100	Kaliyaperumal 2016 [35]
p16	58	90	OC (SCC)	HC	Saliva (Oragene^®^ DNA Self-Collectionkit)	Volume: Kit: Oragene^®^ DNA/Bisulfite treatment; Sigma	MSP	17.2	94.4	Ferlazzo 2017 [39]
MGMT								27.6	92.2	
p16 + MGMT								20.7	-	
ZNF582	94	65	OC (SCC)	HC	Oral rinse (20 mL of mouth rinse solution containing 0.12% chlorhexidine, 20 s)	Volume: 0.4 mL Kit: Epigene Nucleic Acid Extraction, iStat Biomedical/Bisulfite conversion; iStat Biomedical	qMSP	66	84.61	Cheng 2017 [36]
PAX1								68	89.23	
ZNF582 or PAX1								80	78.46	
TRH	42	52	OC	HC	Oral rinse (10 mL 0.9% NaCl, 15 s)	Volume: Kit: QIAamp DNA FFPE Tissue, Qiagen/EZ DNA Methylation-Gold; Zymo Research	qMSP	88.10	92.59	Puttipanyalears 2018 [40]
p16	94	65	OC	HC	Saliva	Volume: Kit: DNeasy Blood and Tissue, Qiagen/EpiTect Plus DNA Bisulfite; Qiagen	MSP	72.2	-	Liyanage 2020 [19]
RASSF1α								68.5	-	
TIMP3								79.6	-	
PCQAP/MED15								79.6	-	
p16 + RASSF1α + TIMP3 + PCQAP/MED15								91.7	92.3	
NID2	43	50/40	OC (SCC)	HC/smokers	Oral rinse (0.9% NaCl, 15 s)	Volume: Kit: Phenol-chloroform extraction/EZ DNA Methylation; Zymo Research	qMSP	79.07	100	Srissutee 2020 [41]
p16	43	40	OC (SCC)	HC	Saliva	Volume: Kit: QIAamp DNA Blood Mini, Qiagen/EpiTect Plus DNA Bisulfite; Qiagen	MSP	44.18	90	González-Pérez 2020 [42]
RASSF1A								23.25	95	
p16 + RASSF1A								53.50	87.57	

Abbreviations: Se, sensitivity; Spe, specificity; OC, oral cancer; SCC, squamous cell carcinoma; HC, healthy controls; MSP, methylation specific polymerase chain reaction; qMSP, quantitative-MSP; OSMF, oral submucous fibrosis, NaCl, sodium chloride.

## Data Availability

Not applicable.
